# Family achievements in struggling with schizophrenia: life experiences in a qualitative content analysis study in Iran

**DOI:** 10.1186/s12888-020-03025-w

**Published:** 2021-01-05

**Authors:** Fatemeh Darban, Roghayeh Mehdipour- Rabori, Jamileh Farokhzadian, Esmat Nouhi, Sakineh Sabzevari

**Affiliations:** 1 Student Research Committee, Razi Faculty of Nursing and Midwifery, Kerman University of Medical Sciences, Kerman, Iran; 2 Nursing Research Center, Kerman University of Medical Sciences, Kerman, Iran

**Keywords:** Family, Achievement, Schizophrenia, Experience, Qualitative

## Abstract

**Background:**

The challenges of living with and taking care of a patient with schizophrenia can lead to positive changes depending on the experiences and reactions of family caregivers. Such changes may directly affect the family performance and the patient’s recovery stage. Present study aimed to explain the positive experiences reported by family caregivers of patients with schizophrenia.

**Methods:**

The present study is a qualitative study of content analysis. Data were collected using semi-structured and in-depth interviews with 15 family caregivers of patients with schizophrenia referring to one of the psychiatric hospitals in Zahedan, Southeast part of Iran. Purposive sampling method was applied and data analysis was conducted using conventional content analysis proposed by Graneheim and Lundman.

**Results:**

Data analysis created a theme entitled “family achievements in struggling with schizophrenia”. This theme included four categories including Developing positive personality traits in family members, Strengthening family ties, developing insight into the life, and social mobility.

**Conclusions:**

The results provided insights that the experience of taking care of patients with schizophrenia led to positive consequences for family caregivers. Thus, it is recommended that psychiatrists or consultants help families rely on positive experiences and share these experiences with families with a newly-suffered patient.

**Supplementary Information:**

The online version contains supplementary material available at 10.1186/s12888-020-03025-w.

## Background

Schizophrenia is one of the most chronic and debilitating psychiatric disorders [1] with a prevalence of 1.4–4.6 per 1000 persons [2]. Early-onset of the disease, long term persistence of the symptoms, and recurrence of the disease cause economic, social, and individual problems. As a result, high therapeutic and non-therapeutic costs are imposed on the family and community, such as losing a job due to illness or taking care of the loved ones [3, 4]. This disease is ranked eleventh among 301 diseases and injuries [5–7]. Families with schizophrenic patients experience grief and have to deal with social stigma and isolation leading to feelings of shame or guilt and affect their physical and mental health [8]. Although most of the consequences of caring for a patient with schizophrenia are negative, some family caregivers have identified more positive and beneficial aspects of this role in recent years [9, 10]. Although little study has been performed in this area, the evidence show that caring for others can result in positive changes in one’s life. Other diagnostic changes can have a positive effect on family members, such as improving the family dynamics, having more social support, and having an outdoor job (for both the patient and the caregiver) [11, 12]. Positive family characteristics such as warmth and cohesion may have a protective effect that helps to improve the symptoms of schizophrenia [13]. In a study conducted by Chen and Greenberg (2004), approximately 70% of the caregivers reported that they became more sensitive towards people with disabilities and 50% felt more empowered internally [14]. Pickett et al. reported that positive assessments of parents’ relationship with the sick child were significantly associated with reduced caregiver burden [15].

Evaluation of the disease consequences depends on the experiences and reactions of the families and patients, which may directly affect the family performance and the patient’s recovery stage [16, 17]. Furthermore, studies show that family structures may vary within different cultural contexts. For example, Weisman et al. (2016) mentioned that the more cohesive the African-American families of patients with schizophrenia are, the less anxious they will be. in [18]. A study indicated that Latin American families managed to accept the patient’s current disabilities and they hope for the future [3]. In Iran, there is a traditional family structure and people with schizophrenia are mostly cared for by their families. In Iranian culture, family ties are important factors in everyday life. The whole family handle their problems and reduce the burden of care [19]. Despite the positive aspects of family care, no study has ever assessed the positive aspects of caring for patients with mental disorders in Iran. The treatment and care team should understand the importance of this issue in providing appropriate care to patients. In other words, these positive family changes can result in medical treatment decisions that enhance disease outcomes. As a result, family caregivers’ experiences should be evaluated comprehensively and deeply. Family caregivers’ experiences can provide the researchers with information about their unique situation and enhance their performance in caring patient [20]. Such experiences can be determined through effective communication and in-depth interviews. Considering the paucity of information about experiences of family caregivers with schizophrenia patients, we decided to explore this issue considering the cultural, religious, and social aspects of the Iranian families.

## Methods

### Study design and setting

This qualitative study was conducted using a conventional content analysis method. This systematic method is applied to describe a phenomenon deeply and release information to determine its patterns and communication processes [21].

This study was performed in Zahedan metropolis, the capital of Sistan and Baluchestan province, southeast of Iran. The residents of this city are of Baloch and Sistani ethnicity. The Baloch speak the Balochi dialect and are Sunni Muslims. The Sistani speak a dialect of Persian and are Shia Muslims [22]. Despite the difference in language and religious beliefs, the residents of this region always tried to meet their shared needs by interacting with each other while maintaining cultural commonalities. They understand each other’s language and live together; get married and have joint representatives in the Islamic Consultative Assembly. Hospitals and the health system employ people from both classes to provide appropriate cultural services. These interactions result in developing a common culture called the culture of Sistan and Baluchestan, which is part of the culture of the southeast of Iran.

The sampling was conducted in a large Educational Psychiatric Hospital in Zahedan, which is the only psychiatric hospital in this city with 100 active beds. The present study was conducted from August 2019 to February 2020.

### Participants

In the present study, researchers used purposive sampling techniques to select participants with different socio-demographic backgrounds to achieve more in-depth information on thei**r** lived experiences. In the hospital, the researcher referred to the inpatient medical records in different wards of the hospital to select several eligible participants. The first researcher collected the patients’ telephone number and address registered in the records and made the necessary arrangements for the interviews. Participants included family caregivers of patients with schizophrenia, who met the following criteria: having a family member with schizophrenia (diagnosed by a psychiatrist), being 18 years old and higher [23], and having favorable physical and mental conditions, living with and taking care of the patient directly, passing at least 1 year from the diagnosis of schizophrenia [24], as well as having the ability to understand and speak Persian or Persian *dialects* - *Baluchi* and *Sistani*. Sampling continued until information was saturated and no new conceptual information was obtained [25]. The researchers prioritized the representation of variations in experience, gender, socioeconomic status, level of education, and ethnicity in the participants.

### Data collection

Data were collected using in-depth, face-to-face, and semi-structured interviews. Initially, the first researcher contacted each participant and scheduled an agreed-upon date and time to conduct the interview. The participant met the inclusion criteria and had consented to participate in the study. All eligible participants were willing to cooperate in the study. The interviews were followed by a topic guide; A list of some helpful questions was prepared by the research team according to the study purpose The Guide of the interview is available in Additional file 1. During the interviews, some questions were also added to the list according to the obtained data. The interview questions were mainly about the family caregivers’ positive experiences in living and taking care of the patient with schizophrenia. The subsequent follow-up questions were gradually raised based on the information provided by the participants for more clarifications. To achieve deeper information, in-depth questions, such as “What do you mean?” Or “Please explain more” were used. A large amount of the data was recollected after the seventh interview and data saturation in the thirteenth interview, but the interviews were continued up to 15 for reassurance. As the participants preferred, the interviews were conducted in the psychologist’s room in the hospital, which is a quiet room. The interviews were performed in Persian (*N*=7) and Balochi dialect (*N*=8). All Balochi interviews were conducted and translated into Persian before data analysis by F. D (the first author). F. D is a native Balochi and Persian speaker and familiar with a combination of Balochi and Sistani culture. The interviews lasted 45–90 min.

All interviews were recorded with the consent of the participants. The recorded files were transcribed verbatim; even the participants’ nonverbal gestures and body movements were mentioned in the transcriptions. The interview process was evaluated by the second and third researchers, who are experts in qualitative study.

### Analysis

Data collection and analysis were performed simultaneously. The MAXQDA.18 used to facilitate organization and comparison of the data.

The transcription of each interview was reviewed several times. The qualitative data content analysis process was performed according to the method proposed by Graneheim and Lundman, including writing the entire interview, reading the entire text of interviews several times to achieve a general understanding of its content and immersion in the data, determining semantic units and summarizing them, extracting the primary codes, classifying the similar primary codes under the same subcategories, classifying similar codes under more comprehensive categories, extracting latent and manifest concepts from the data, and formulating the final themes [21]. To this end, after preparing the transcriptions, each text was reviewed several times. Later, the semantic units were identified based on the research questions and appropriate codes were written for each semantic unit. As shown in Table 1, the preliminary codes were categorized and labeled based on their conceptual similarity (subcategories). The subcategories were compared and placed under the main categories, which were more abstract (categories). The main categories were categorized under a more abstract concept (theme).

**Table 1:** a sample of formation of a category

Category	Subcategories	Examples of Codes	Examples of semantic units	Examples of quotations
Strengthening family ties	Increasing dependence and affection among family members	-Understanding the love in the family - Getting closer together -Understanding dependence	-Perceiving the intensity of a father’s interest in his children after a child was ill -The parents’ getting closer after the child’s disease -Perception of the family members’ love as a result of the disease	“Now, I understand how much my husband loves our children. When one of our children was hospitalized, my husband had a stroke and was hospitalized too ... Due to this, I am more interested in my husband than before; I think we are closer than before”.
	developing family members’ empathetic participation in caring patient	Participating in treatment Collaborating on care Collaborating on tasks	- following the treatment process of the patient by All family members -Cooperation of married children inpatient care -Patient care by all family members	- My sister takes my mother to the clinic every month; she keeps the date of visits. Physicians tell us about the doses of her medicines and I give her the medicines. - When we have some other things to do, my sister takes my mother to her house and her husband is very considerate of my mother.
	Increasing support among family members	-Supporting others - Supported by others - Trying to support the caregiver	-Family members’ support of younger children -Children’s support for the father of the family -The children’s efforts to reduce the burden on the father of the family	“My father and older siblings took care of the younger children”. “As we got older, we paid a lot of attention to my father and helped him to reduce the burden on his shoulders”.

All extracted codes and categories were reviewed and approved by the second and fifth authors of this study. The initial extracted codes were reduced by continuous data analysis and comparison; finally, the categories and subcategories were abstracted.

Lincoln and Guba criteria (credibility, dependability, confirmability, and transferability) were used to ensure The data trustworthiness [26]. To ensure the results credibility, participants were asked to confirm the extracted codes from the interview and resolve the contents on demand (member check). Data-source triangulation from interviews with family caregivers with variety in relationship with patient, ethnicity and religion established credibility. Regarding confirmability of the findings, all texts of the interviews, codes, and categories were reviewed and confirmed by the second, third, and fifth authors of this study (peer check) as well as a faculty member outside the research area (faculty check). To ensure the dependability of the results, all stages of the study were recorded. Participants were selected by maximum variation sampling in terms of ethnicity, level of education, religion, economic status, relation to the patient, and social class, *which enhanced the transferability* of the study.

## Results

A total of 15 interviews were conducted with the family caregivers of schizophrenic patients about the positive consequences of schizophrenia for the family. Participant characteristics are shown in Table 2. The family caregivers included 11 females and 4 males with monthly income ranging from 10 to 50 million rials. 8 of them were Baloch and 7 Sistani ethnicity (Table 2). Their patients included 6 females and 9 males aged 26–56 years, and a 5–22 years of disease duration. Two patients had a university degree and the others had a high school or lower education. All but one patient were Unemployed.

**Table 2:** Participants demographic characteristics

No. of Caregivers	Age	Marital status	Educational status	Religion	Relationship with patient	Occupational status	duration of the disease(year)	Years of caregiving
1	42	Married	Middle school	Islam/Shia	Sister	Unemployed	8	8
2	40	Married	Primary school	Islam/ Sunni	Spouse	Paid employment	22	22
3	33	Divorced	Middle school	Islam/ Sunni	Brother	Paid employment	9	2
4	31	Married	No formal education	Islam/ Sunni	Spouse	Paid employment	9	9
5	32	Single	University	Islam/ Sunni	Uncle	Full time employment	6	6
6	30	Single	High school	Islam/Shia	Sister	Paid employment	5	5
7	45	Married	Primary school	Islam/ Sunni	Mother	Unemployed	5	5
8	40	Married	Primary school	Islam/Shia	Half-daughter-in-law	Paid employment	8	8
9	65	Married	University	Islam/Shia	Mother	Retirement	8	8
10	43	Married	University	Islam/Shia	Spouse	Paid employment	10	10
11	32	Married	University	Islam/ Sunni	Son	Full time employment	18	10
12	25	Single	High school	Islam/ Sunni	Daughter	Unemployed	15	10
13	24	Single	University	Islam/Shia	Daughter	Student	20	8
14	55	Married	Primary school	Islam/ Sunni	Spouse	Full time employment	20	20
15	48	Married	Primary school	Islam/Shia	Spouse	Unemployed	18	18

As shown in Table 3, Data analysis resulted in a theme entitled “family achievements in struggling with schizophrenia”, which included four categories and 17 subcategories: Developing positive personality traits in family members (being able to express emotions easily, being *stronger in the face of life* problems, making greater intellectual developments than peers, being patient, having enhanced communication skills, achieving independence faster, and having increased ability to understand others); Strengthening family ties (increasing dependence and affection among family members, developing family members’ empathetic participation in caring patient, and increasing support among family members); Developing insight into the life (understanding the importance of mental health, changing mindset from comfort to peace in life, feeling the presence of superior power in life); and Social mobility (a flip to change lifestyles, acquiring and enhancing capabilities, Economic dynamism, Dynamism in social services).

**Table 3:** Theme, Categories and subcategories extracted from the data

Theme	Categories	Subcategories
Family achievements in struggling with schizophrenia	Developing positive personality traits in family members	being able to express emotions easily
		being *stronger in the face of life* problems
		making greater intellectual developments than peers
		being patient
		having enhanced communication skills
		achieving independence faster
		having increased ability to understand others
	Strengthening family ties	increasing dependence and affection among family members
		developing family members’ empathetic participation in caring patient
		increasing support among family members
	Developing insight into the life	Understanding the importance of mental health
		Changing mindset from comfort to peace in life
		Feeling the presence of a superior power in life
	Social mobility	A flip to change lifestyles
		acquiring and enhancing capabilities
		Economic dynamism
		Dynamism in social services

### Developing positive personality traits in family members

Based on the findings, living with and caring for a patient with schizophrenia caused significant changes in people’s daily lives. Adaptation to these changes resulted in the development of positive personality traits in people who were living with these patients. According to the participants’ experiences, seven subcategories were obtained concerning the development and growth of positive personality traits in the patient’s family members.

#### Being able to express emotions easily

Based on the participants’ experiences, the challenges of living with a patient with schizophrenia caused them to show their emotions (e.g., happiness or sadness) more openly and to react emotionally to small events. Their feeling of compassion to the patient had led them to show this feeling to others.

“*My heart goes out to my mother. Because of her illness, I became so empathetic to others. I have suffered so much that I become happy with good news. I think I am much more compassionate than before; watching a sad movie makes me move to tears*”. (C13).

In fact, the disease caused the family caregivers to become more sensitive to life events and react to small changes:

“*My sister’s illness has made it much easier for me to express my feelings than before. For example, if one of my stuttering students talks in full sentences without stutters, I will be so much happy and excited, so that even his mother will not show that much excitement”*. (C6).

#### *Being Stronger in the face of life* problems

The problems raised by living with a schizophrenic patient strengthened the family caregivers’ self-confidence. They believed that these difficulties made them stronger so that they could handle other problems in life.

*“Those hard days made me strong and impassive. As strong as iron in the face of these problems. When I encounter a problem, I consider it nothing compared to those hard days*”. (C15).

In case of family member’s disease, such as a spouse, hiring caregivers to carry the burden of life by accepting new roles and tasks is required. They experienced new situations throughout the care path, which made them more prepared for the future events.

“*When my husband got neurological disorders, I had to manage my children, so that I would not need anyone. So, I stayed as solid as a rock. And did not give up”.* (C2).

#### Making greater intellectual developments than peers

Children of patients with schizophrenia were severely affected by the disease so that their needs are not met appropriately. Based on the children’s gender, some roles may be assigned to them, which enable them to reach intellectual maturity earlier than their peers.

*“Comparing myself to my peers, I notice that I am more mature with than them regard to the surrounding issues. Sometimes, my friends tell me: ‘you are very wise and understand beyond your age”.* (C11).

“*In other words, my mother’s disease helped my mind grow faster; that is, I became more mature”.* (C12).

#### Being patient

One of the positive aspects of having a family member with schizophrenia was patience. In such families, patience resulted from caring for the patient is expanded through the life. A participant stated that she became much more patient in social interactions than the past and could tolerate inappropriate behavior of others much easier.

“*I became much more patient than before; struggling with him increased our endurance and patience more than before. I realized that my patience had increased in dealing with others”.* (C5).

Participants noted that this patience was a reward from God. They got surprised that they had achieved such strength and endurance, as the result of peaceful behavior with the patient.

“*God has given me a patience; in my family, I am famous for being patient. Sometimes, I wonder how much patient I am. In dealing with my husband’s behaviors, I learned that I should be silent when he is angry ... this increased my patience”.* (C10).

#### Having enhanced communication skills

The development of communication skills was one of the family’s achievements from living with schizophrenic patients, which was noted by participants, especially female caregivers. The illness of a family member, especially the spouse and father of the family, *resulted in the loss or decrease of income*. So, other family members, such as the patient’s spouse, were required to work outside the home to pay for the expenses. Outside jobs provided the opportunity for individuals to interact with many people from different social backgrounds and strengthened their communication skills.

*“From the onset of my husband’s disease, I have been working in different people’s homes; for example, one was a doctor and one was a businessman. Every family was different and commuting to their homes helped me to learn how to speak well and communicate easily with others. As a result, I do not feel nervous while talking to others anymore”.* (C2).

#### Achieving independence faster

Children of a parent with schizophrenia had to participate in household chores; they even needed to cooperate in caring for the patient. Such early cooperation and tasks allowed them to achieve independence sooner than their peers and strengthened their sense of responsibility.

“*I stood on my own two feet much earlier than my friends and became independent ... I kept the wolf from the door so that I do not put my burden on my siblings’ shoulder. In other words, my mother’s illness made me grow up. My life’s route was so that I had to grow up and become independent very quickly”.* (C13).

#### Having increased ability to understand others

Incidence of the disease in a family exposed the family members to the judgments of others. Lack of understanding by others caused feelings such as sadness, embarrassment, and stigma in family members. Experience of these negative feelings caused the patient’s family caregivers to try to understand others in similar situations and avoid misjudgments because they have already been victims of negative perceptions of others.

“*This disease has taught me never to judge others and their lives or to make fun of anyone. This reminds me of my life, I know that judging and making fun of others can have harmful and negative consequences”.* (C1).

### Strengthening family ties

This category showed that when one family member had schizophrenia, the other family members got involved in caring for the patient to relieve the burden. As a result of this cooperation, the family ties strengthened.

#### Increasing dependence and affection among family members

The reaction of family members to the suffered member strengthened their love to each other. In present study, the father of a patient had a heart attack when his son was hospitalized in a psychiatric hospital. The presence of a disease in the family made the family members realize how much they loved each other.

“*Now, I understand how much my husband loves our children. When one of our children was hospitalized, my husband had a heart attack and was hospitalized too ..., for this reason, I love my husband more than before; I think we are closer than before*”. (C7).

Members of the patient’s family considered the illness as a common pain, which required empathy between family members, a feeling that brought family members together, and increased love between them.

“*My son’s illness has brought me and my husband closer. This pain has made us more dependent on each other to take care of our son ... Now my son is in a condition that we need to be more empathetic, and that is what happened”*. (C9).

#### Developing family members’ empathetic participation in caring patient

The disability caused by schizophrenia may be so severe that the patient may not be able to perform personal daily tasks and hygiene self-care such as bathing, dressing, and eating, and need the help of others. When family members were aware of the patient’s disabilities and knew that no one could take care of the patient alone, they felt obligated to participate in housework and patient care.

“*From the onset of her disease, my husband cooperates in housework more ... For example, when my husband gets up early in the morning to go to work, he feeds her breakfast. He also helps me give her medications, take her to the doctor. Now, we are taking care of her together*”. (C8).

Participation in caring patient was not confined to family members, but also it can extend to married children who live separately and can cooperate with the family to reduce the stress and burden.

“*My brother and sister are married and do not live with us, but they still help us take care of my sister; they take her to the doctor, hospitalize her, and even pay for her hospitalization. They visit and help us more than before since my sister got ill”*. (C6).

#### Increasing support among family members

Due to the lack of social support resources in Iran, families of patients with schizophrenia experience severe financial, emotional, and psychological stress. Experiences of family caregivers in present study showed that in such situations, family members provided the role of financial, emotional, and psychological support for the caregiver.

*“Since my husband got sick, my children supported me so much. With their support, I was able to go to the university and get two bachelor’s degrees; my children helped me a lot ... my family also supported me and my children more than before”.*(C10).

Supporting caring parents by family members helped them to adapt to the changes caused by schizophrenia.

*“My children are very attentive to me, even my older children support their younger siblings more than before. My children’s support made me able to cope with my wife’s illness”.* (C14).

### Providing insight into the life

Based on the participants’ experiences, living with schizophrenia patients provided them insight into life. As a result of illness, they tried to change their attitude toward life, think deeply, and pay attention to what they had neglected before the illness.

#### Understanding the importance of mental health

The experience of families living with schizophrenics made them realize the importance of mental health in life and worry about their health. One of the *causes of* distress *among caregivers* was their qualifications to help the patient.

“*Since my brother became ill, I feared developing the disease. I was obsessed with it for a while, so I went to a psychiatrist and now I am under control. Because I know if I get sick, there is no one to take care of my brother; so, I considered my health”.* (C3).

The positive and negative symptoms of schizophrenia patients and their destructive behaviors had destructive effects on the patient and caregivers. So, the participants tried to avoid what harms their mental health, such as getting upset and nervous. They avoided trivial issues and considered their mental health as one of the most important aspects of health that should be protected.

“*Since my sister became ill and I saw her behaviors, I realized that maintaining mental health is crucial. I am more concerned about my health and my children’s health. I try not to get upset by little things”.* (C1).

#### Changing the mindset from comfort to peace in life

Losing family peace due to stressful factors caused by schizophrenia helped the participants to conclude that having peace in life was much more valuable than gaining wealth, facilities, and money. They believed that comfort was effective under the shadow of peace.

“*Even though I am old, the disease has changed my mindset. Previously I wanted my children to have good jobs and good incomes; that is to say, I was worried about their comfort. Now, I believe that peace in life is much more important than money”.* (C9).

Participants stated that the presence of this disease changed their attitude towards life events; so, they tried to take life easier.

“*Now, I try to make the events easy, I think they are not worth being upset, I should not grieve for them and ruin my life, I can handle them”.* (C12).

#### Feeling the presence of superior power in life

Spirituality and closeness to God were among the other positive experiences of family caregivers with a schizophrenic patient. Participants believed that taking care of the patient was a blessing in their life and a reward from God. In fact, they believed that God bestowed them rewards for taking care of a patient.

“*This is how God gives me sustenance. Since she is living with us, our sustenance has been growing. She has sustenance, when I take care of her, God gives me more sustenance. I do not mean the money, but the blessings”.* (C8).

The presence of the disease led most family members to engage in religious activities and participate in religious ceremonies. They noted that this closeness to God was impacted by the illness of a family member since they felt God’s presence in their lives more than ever before.

*“With my mother’s illness, I became much closer to God, which means I pray a lot. I vow and attend religious ceremonies. Maybe if my mother was not sick, I would not be so close to God. I feel God has taken my hand in some situations and helped me”.* (C13 (.

### Social mobility

As a result of the illness of a family member, especially the father or spouse, the family lost its social and economic position. The experiences of some participants showed that the family members tried to reach their previous social status, improve their social class, and build their lives.

#### A flip to change lifestyles

The suffering in caregivers’ life made them decide to change their lives, build their own lives, and compensate for the damage done to the family.

*“When my husband fell ill, I said: ‘I cannot just sit around and do nothing. I have two children and I have to build my life.’ So, I started to change”.* (C10).

The children of the patients were also determined to choose a good path in their lives. They tried to be beneficial to society; so, they had higher levels of motivation to study and find a suitable job.

*“I got my diploma ... I went and bought an organ ... One day I came to myself and said: ‘what do you want to do?’ At that moment, I decided that I should change my life and serve the people around me ... I sold my organ and bought law books; I told myself that I must go to law school and I should be a member of the parliament in the future. Fortunately, I entered the university the same year”.* (C11).

#### Acquiring and enhancing capabilities

The participants’ experiences showed that family members of patients decided to work harder to support themselves. As a result, they began to improve their previous skills or learn new ones based on their working situation. However, if the family head was not sick, they would never think about acquiring or improving their skills.

“*My husband’s illness has made me learn live my life. I know needlework; so I went to my mother’s house since she does needlework too... During this time, I saw different models and learned them; I did not know them before”.* (C4).

To forget about the problems of living with the patient, some participants tried to entertain themselves with their favorite activities, such as exercising, sewing, going to university, etc. Such activities improved family members’ skills.

“*To entertain myself and forget about my sick husband, I was preoccupied with my favorite activities. I continued my studies, got a diploma, and then got two bachelor’s degrees. I started a sewing school, established a gym, and continued my favorite sport ...”* (C10).

#### Economic dynamism

According to the participants’ experiences, family responsibilities led them to pursue income-generating activities; their efforts to support family expenses improved their economic conditions.

“*I could manage my life even better than when my husband was not sick. Thank God my income was not bad. I helped my daughter and son to get married. I work and pay for my husband’s expenses. Once a year, I hospitalize him”.* (C2).

The experience of living with a patient with schizophrenia, which is a chronic and debilitating disorder, has taught the participants to manage their lives financially and provide themselves with the necessary welfare amenities. They learned not to wait for help from others. So, they performed income-generating activities.

*“I have learned that I have to work and pay for my children anyway. To this end, I accept needlework orders. I have a little income, but it is better than before since I do not ask others for money”.* (C4).

#### Dynamism in social services

According to the participants, dealing with the suffering of the disease and observing its negative effects made them move beyond the family conditions and decide to serve other people in the community.

*“I sought to establish a charitable school on the outskirts of the city ... or I pursued a lot to get the privilege of a facilitation office there ... I owe all this success to the hardships I endured because of my father’s illness”.* (C11).

The patient’s activities before the disease were a motivation for the participants to help others.

“*After my sister’s illness, when we saw that she could no longer go to the local mosque, my sister and I went there. When we do something for the people; well, God will take our hand. We could collect donations for the recent flood victims”.* (C6).

## Discussion

According to the results, family caregivers of the patients with schizophrenia experienced positive consequences of taking care of the patient, which are a result of their struggle with this disease. The positive consequences included four categories included developing positive traits in family members, strengthening family ties, developing insight into life, and social mobility. Shiraishi and Reilly (2019) in a qualitative meta-summary, investigated the positive and negative consequences of schizophrenia in family caregivers. They concluded that family members of a person with schizophrenia experienced a series of traumatic events at the onset of the disease. Subsequently, they faced with a continuous cycle of care. In this cycle, they experienced negative effects of the disease, including insecurity, loss of life expectancy and health, decreased personal and social resources, stigma, etc. Experiences in these conditions led to the emergence of positive aspects of care and values such as family solidarity and personal growth [5]. Based on the participants’ experiences, the sufferings that families endure in taking care of the patient have led to positive consequences and changes in their lives.

In a study by Wiens and Daniluk (2017), mothers of children with schizophrenia experienced personal changes during child care. These positive changes included a deep sense of awareness of others’ needs and sensitivity, less judgment, and higher levels of compassion. They believed that dealing with the child’s illness increased their patience and endurance [27]. These results are consistent with those of present study on the development of positive personality traits. The results of some studies showed the presence of a schizophrenic patient in the family caused personal growth in the caregivers [28–31], which confirms the experiences of our participants. This growth is not a goal, but a response to what life is all about. In other words, this growth is a valuable part of the human experience that helps them cope with the ongoing challenges of life [32]. Increased self-confidence [31, 33, 34], easier expression of emotions [31, 35, 36], and increase of patience and tolerance [27] were positive consequences of the caregivers’ personal growth in various studies, which are consistent with the results of the present study.

Based on the participants’ experiences, strengthening family ties through family caregivers’ support, participation in caring patient, and increased love for each other were the family achievements in dealing with the disease. Some scientist (Mizuno et al. (2011), Barnable et al. (2006), Sethabouppha and Kane (2005), and Wiens and Daniluk (2009)) reported an increase in love and affection among family members after the incidence of the disease [27, 29, 36, 37], which confirm results of the present study. An increase in family cohesion was also reported as positive experiences of the family members with schizophrenic patients in other studies [27, 33, 38], which are similar to the results of our study. When a family member develops schizophrenia, the difficulties caused by the disease may disrupt the family. If family members stay together and share care responsibilities, they become closer to each other and the primary caregiver feels less burden and becomes stronger [14].

Social mobility in terms of economic, social, and professional empowerment was another result of the present study. Furthermore, researchers in several studies reported that increased levels of knowledge and skills were the positive consequences of caring for a patient with schizophrenia [31, 34, 38]. In our study, participants tried to build their lives by learning new skills, upgrading their previous skills, and seeking to gain a better social and professional position.

The development of insight into life was another positive consequence of the disease mentioned by our participants. Chen et al. (2004) conducted a quantitative study and concluded that caregivers gained new insights into their lives in the process of caring for the patient with schizophrenia [14]. Some researchers (Sethabouppha and Kane (2005) and Wiens and Daniluk (2009)) indicated that unforeseen challenges of living with a patient led people to appreciate life more and perceive the meaning of love and affection better [27, 36].

The results suggest that mental health professionals should help family caregivers decide to better respond to their challenging roles and increase their sense of accomplishment and personal growth. In the present study, we reported a set of care benefits for family caregivers that could be used as a theoretical method to design appropriate interventions for families and their patients. Interventions should target the use of internal resources and family abilities such as personal characteristics, family relationships, religion and spirituality, and socioeconomic integration of family members aimed at facilitating the process of living with schizophrenia patients. In this way, it is possible to cause positive changes for family caregivers and patients.

Participants had a variety of shared experiences of a positive life with schizophrenia patients and caring. However, each experience is unique, and each family caregiver could only understand the cultural and religious context in which he or she lives. Therefore, we suggest that health care interventions be appropriate to the religious, cultural and ethnic structure of the families to strengthen the internal strengths in the families of patients with schizophrenia. More detailed studies are recommended to evaluate the results of the present study in Iran and other cultures, and contexts and assess how the relationship between the patient’s recovery and positive outcomes perceived by family caregivers.

### Limitations

The present study was performed in the southeast part of Iran, so the generalization to other cultural groups needs more studies in other cultural groups. Because families take care of patients at home, observing all behaviors of family members at the desired times was not possible. The researcher tried to control this limitation by long engaging with the family members and gaining their trust so that they could express their behaviors freely.

## Conclusions

The present study provides some evidence about the positive consequences of living with a schizophrenic patient based on the experiences reported by caregivers. In fact, the challenges of life and family experiences in caring for the patient have led to positive changes in family perceptions. As a result, investigating valuable experiences of the family members in caring for the patient and examining their strengths can help the researchers to design and develop family interventions for better patient care.

## Supplementary Information

**Additional file 1.**

## Data Availability

The transcripts from which this script was written are available on request from the corresponding author.
