# Supplementary material for: Family achievements in struggling with schizophrenia: life experiences in a qualitative content analysis study in Iran
Source: BMC Psychiatry. 2021 Jan 5;21:7. doi: 10.1186/s12888-020-03025-w (PMC7786938; doi:10.1186/s12888-020-03025-w)
Supplement: Supplementary file 1 — Additional file 1. [file 12888_2020_3025_MOESM1_ESM.docx]

**The topic guide for interview:**

- Demographic characteristics of caregivers and patients

- Please tell us about your experiences of living with a schizophrenic patient and caring for him

- What factors helped you to endure these conditions?

- What positive changes have been made in your life as a result of living with a schizophrenic patient? (Thoughts/feelings/ Behaviors / Performance / Activities / Communication / Emotions / Personality Traits …)

- What have been some of your most rewarding experiences in your caregiving role?

- How do you feel about the caregiving role?

- What positive changes have taken place in your life after patient care compared to before?

- What resources or support have you obtained?

- Can you explain your views on living with your patient and caring for him?

- Please explain to us about the positive impact of living with patient (and caring for patient) on your skills and economic activity?

- Please explain to us about the positive impact of living with patient (and caring for patient) on your social activity?

- Please explain to us about the positive effect of living with patient (and caring for patient) on your attitude and mindset?

- Please explain to us about the positive impact of living with patient (and caring for patient) on your family communication?
